# Understanding the Basics of NGS: From Mechanism to Variant Calling

**DOI:** 10.1007/s40142-015-0076-8

**Published:** 2015-09-04

**Authors:** Dale Muzzey, Eric A. Evans, Caroline Lieber

**Affiliations:** Counsyl Inc., 180 Kimball Way, South San Francisco, CA 94080 USA; Joan H Marks Graduate Program in Human Genetics, Sarah Lawrence College, Bronxville, NY USA

**Keywords:** Next-generation sequencing (NGS), Variant calling, SNP/indel calling, Del/dup calling, Read depth

## Abstract

Identifying disease-causing mutations in DNA has long been the goal of genetic medicine. In the last decade, the toolkit for discovering DNA variants has undergone rapid evolution: mutations that were historically discovered by analog approaches like Sanger sequencing and multiplex ligation-dependent probe amplification (“MLPA”) can now be decoded from a digital signal with next-generation sequencing (“NGS”). Given the explosive growth of NGS-based tests in the clinic, it is of the utmost importance that medical practitioners have a fundamental understanding of the newest NGS methodologies. To that end, here we provide a very basic overview of how NGS works, with particular emphasis on the close resemblance between the underlying chemistry of Sanger sequencing and NGS. Using a pair of simple analogies, we develop an intuitive framework for understanding how high-confidence detection of single-nucleotide polymorphisms, indels, and large deletions/duplications is possible with NGS alone.

## Introduction

Assembly of the first human genome sequence consumed 12 years and cost nearly $3 billion [1, 2••, 3, 4]. The effort involved hundreds of researchers around the world and was a tour de force of the “first-generation” Sanger sequencing technology developed in the 1970s. Unfortunately, by the end of the Human Genome Project in 2002, this mature sequencing technique was already operating at nearly peak efficiency, making it totally unsuitable for scaling up to the task of sequencing millions of patients’ genomes quickly and affordably. Therefore, in order for the theoretical promise of personalized genomic medicine to become a clinical reality, a quantum leap in sequencing technology was required.

Remarkably, not even 15 years after decoding the first human genome, NGS techniques [5] now enable the sequencing of an entire human genome in a single day for around $1000. These advances have allowed NGS-based tests to enter the clinic, where they are an exponentially growing presence in carrier screening [6••], testing for fetal aneuploidies [7, 8, 9•], detecting the presence of rare diseases [10], and assessing both the risk and existence of cancer [11, 12••]. The clinical utility of an NGS-based test stems from its ability to confidently identify the differences between a patient’s genome and the reference genome. Such genomic differences—called “variants”—fall into two classes: (1) changes to the DNA sequence, e.g., the single-nucleotide polymorphisms (“SNPs”) and short insertions/deletions (“indels”) in the *CFTR* gene that can cause cystic fibrosis [13], and (2) large deletions/duplications (“del/dups”, a.k.a., “copy-number variations” or “CNVs”), e.g., the whole-gene deletions of *HBA1* and *HBA2* that largely determine the presence and severity of alpha-thalassemia [14]. Here we discuss how NGS is exquisitely capable of revealing both types of variants in patients’ genomes.

## How Does NGS Work?

The term “NGS” does not denote a single technique; rather, it refers to a diverse collection of post-Sanger sequencing technologies developed in the last decade. These innovations include sequencing-by-synthesis [15], sequencing-by-ligation [16], ion semiconductor sequencing [17], and others. However, because the predominant technique used in genetic medicine today is the sequencing-by-synthesis approach employed by Illumina devices, here we use the term NGS to refer specifically to Illumina-style sequencing.

**Fig. 1:**
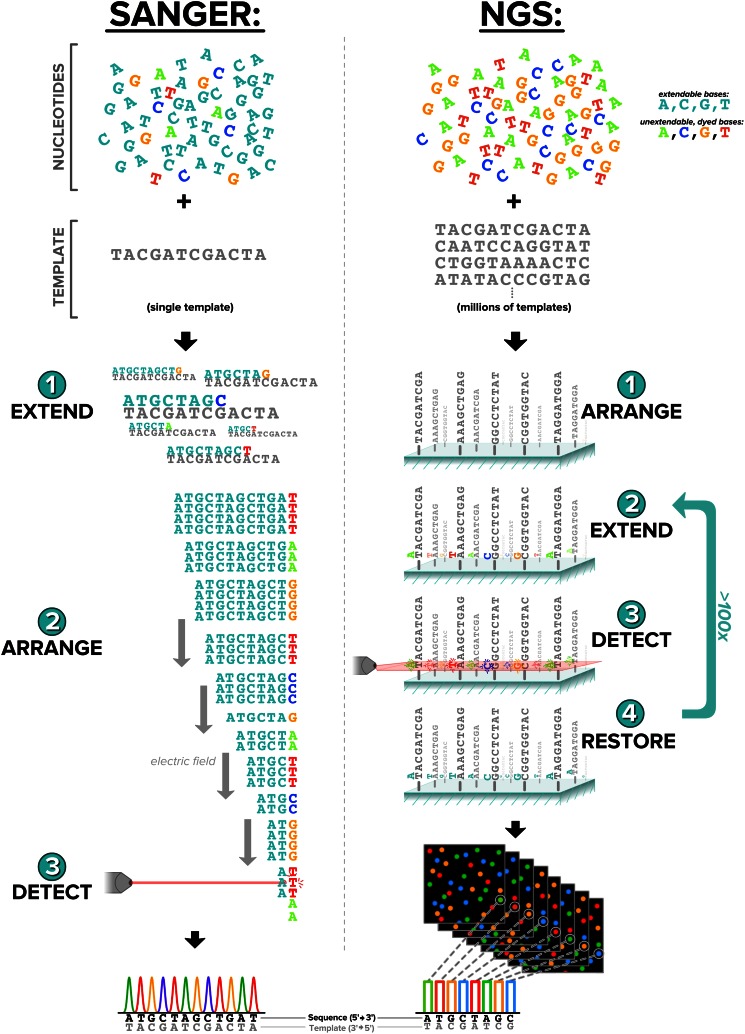
NGS is a slightly modified, digital, and vastly scaled-up implementation of Sanger sequencing. In both methodologies, a polymerase copies template molecules by incorporating nucleotides from a pool, that is, either partially (Sanger) or entirely (NGS) composed of dyed and unextendable bases. Extension, arrangement, and detection are shared steps in both protocols but occur in different order, with NGS alone having a restoration step that converts bases to the undyed and extendable form

Even though NGS is largely displacing Sanger sequencing in molecular diagnostics [18, 19], the two technologies share a common origin that dates back millions of years: both repurpose the DNA replication machinery that copies DNA during every cell division. In a conceptually simplified form, DNA replication requires only three types of molecules: a template strand, free bases, and a polymerase enzyme that links the free bases together one-at-a-time into a new strand complementary to the template (Fig. 1, top).

The key innovation that transforms DNA replication into the DNA-sequencing strategy at the core of both Sanger and NGS is the use of unextendable, fluorescently labeled modified bases. There are four different colors of modified bases for A, T, G, and C (Fig. 1, top). In Sanger sequencing, only a small percentage of bases are modified, whereas in NGS, all available bases are modified. In both sequencing techniques, when polymerase incorporates a modified base into the copied strand, extension of the new strand stops, and, critically, this newly terminated strand is uniquely colored to reflect its most recently added base.

The fundamental challenge for the sequencer, then, is to organize molecules such that their fluorescence signal is interpretable. In Sanger sequencing, an ensemble of DNA molecules—all originating from the same position on the template but having different size due to termination at different positions—are arranged in an electric field, which separates them by size because DNA is negatively charged [20•]. As the molecules migrate in the presence of the electric field, they flow past a detector that registers the fluorescence intensity and color, yielding a series of peaks that can be mapped directly to a DNA sequence.

Rather than exploit size separation to arrange the fluorescent molecules, NGS uses positional separation: millions of different template DNA strands bind to discrete positions on a glass slide and remain fixed at the same position throughout the entire sequencing reaction. Each template is then extended by a single modified base, and a microscope captures an image that resolves both the position of each template on the glass as well as its fluorescent color and intensity (for clarity, an amplification step is omitted from Fig. 1 whereby each template is copied nearby on the glass slide such that fluorescence signal is amplified). Next, in a step unique to NGS, the modified bases are converted to regular bases, such that they become both extendable and non-fluorescent. This restoration process primes them to undergo subsequent rounds of single-base extension and imaging. At the end of a sequencing run with *n* imaging cycles, the fluorescence color at each template position in each image is mapped to a base (i.e., A, T, C, or G). The bases from a single template position are concatenated to yield a DNA sequence of length *n*, called a “read.” Interestingly, although initial NGS read lengths were <100 and trailed behind Sanger’s typical 400- to 500-base sequences, newer NGS machines can match or exceed the length of Sanger-generated sequences.

Our discussion thus far illustrates that both NGS and Sanger are extremely similar in that they perform a few basic and common steps: extension of DNA molecules one base at a time in the presence of modified bases, arrangement of those molecules (either by size in Sanger or by location with NGS), and detection of fluorescence. The two techniques are distinguished, however, by the order of these steps and the inclusion of the restoration step in NGS. Although these differences are subtle, they have a tremendous impact on throughput. While a Sanger reaction returns a single DNA sequence, a typical NGS experiment can yield more than 250 million unique reads. To gain some perspective on this huge number, consider that 100 Sanger sequencers running around the clock would need about 3.5 years to sequence the human genome, whereas a single NGS machine can do the same in a little more than one day.

## How Does NGS Allow Confident SNP and Indel Identification?

**Fig. 2:**
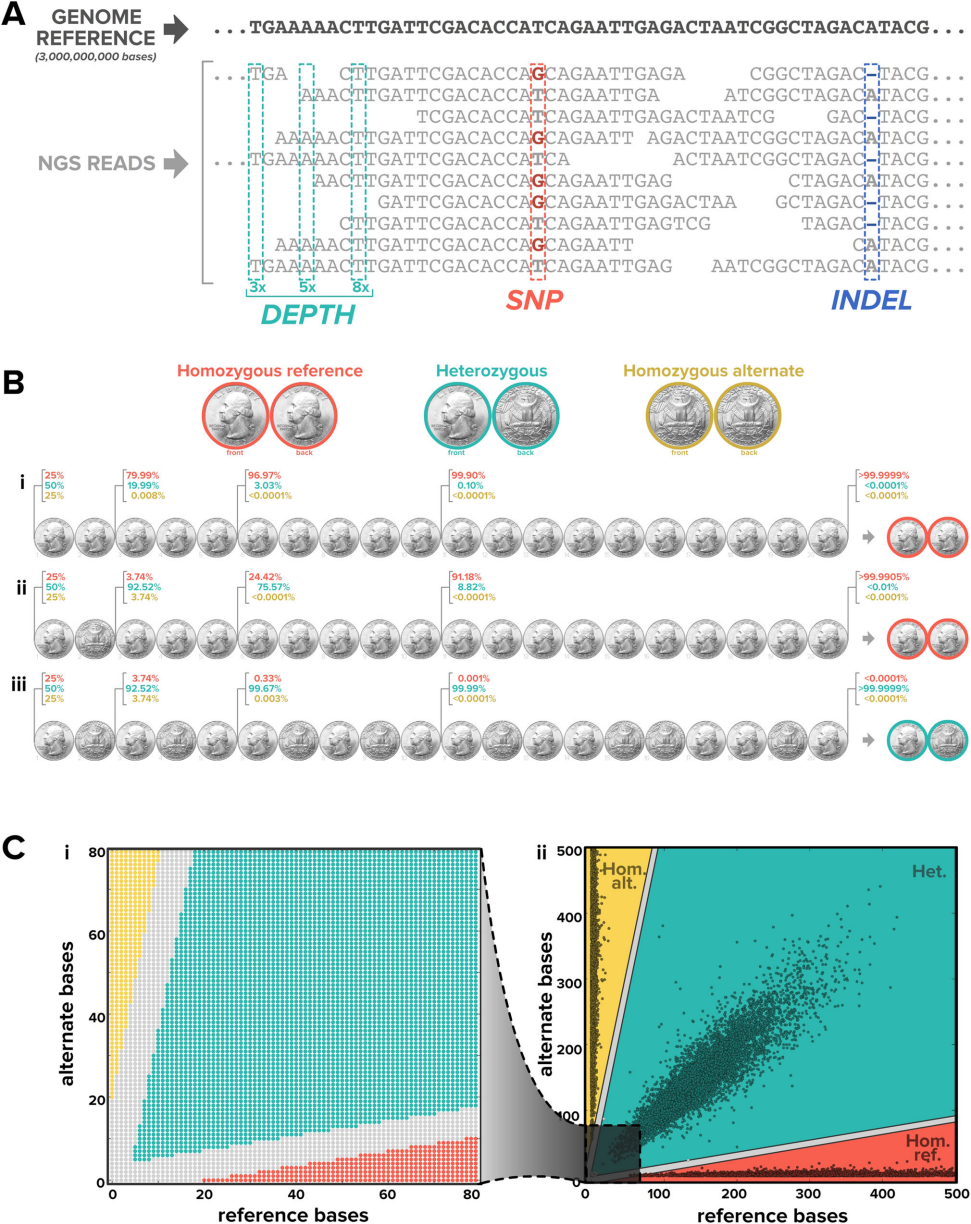
High-confidence SNP and indel calls possible from NGS data with >20× depth. **a** SNPs and indels are conspicuous from NGS data after the reads (*gray*; each read is 28 bases long) are aligned to the reference genome (excerpted in *black*), and the confidence of each call depends on the depth at that position. **b** The three potential genotypes for a simple diploid variant are represented as different types of coins (*top*). A referee who lies about the coin-flip outcome 1 % of the time reports the results of 20 successive flips for three different coins (*i*–*iii*); the probability that the referee selected each type of coin is indicated after 2, 5, 10, and 20 flips, with the coin at right being the one with maximum probability. The probabilities indicated before the coin is flipped assume the coins model a genomic variant with 50 % minor allele frequency (“MAF”). **c** (*i*) Call confidence as a function of respective read depth for reference and alternate bases is shown, where *gray* regions have confidence <99.9999 %, and the three-colored regions have >99.9999 % confidence in homozygous reference (*red*), heterozygous (*green*), and homozygous alternate (*yellow*) calls. (*ii*) Each point shows the reference-versus-alternate read depth across sites with MAF ≥45 % in a typical targeted NGS experiment

Although NGS yields a staggering amount of sequencing data, its ability to transform genetic medicine relies on identifying clinically relevant variants with high confidence. Variant identification begins with alignment of NGS reads to the human genome reference sequence [21], depicted in Fig. 2. Although there are three billion bases in the human genome, reads of length ≥25 are typically sufficient for unique alignment, even allowing for mismatches or gaps. The number of reads that align at a given position is called the “depth” or “coverage.” Variants are simply deviations from the reference sequence. For instance, heterozygous SNPs (Fig. 2a, red) manifest as positions where approximately half of the reads match the reference, and the other reads differ from the reference. Indels shorter than the read length are similarly conspicuous, with a nearly 50/50 split between reference reads and gapped reads.

A recurrent source of confusion in the field is how much depth is required to make confident SNP and indel calls. For instance, is 5× depth enough? Is 50× needed? Does 1000× depth give much better performance than 100×?

To give insight into these questions, we use an analogy in which coins represent genotypes. Suppose we have three coins: one has two heads, one has a head and a tail, and one has two tails. Imagine that a referee picks a single coin, repeatedly flips it while announcing heads or tails, and, after a number of flips, asks you to say which coin was selected. To make the game slightly more challenging, suppose the referee lies 1 % of the time and reports heads when the coin lands on tails, and vice versa. This scenario is highly analogous to the challenge of variant identification from NGS data: each of the three coins is a possible genotype (two heads is “homozygous reference,” a head and a tail is “heterozygous,” and two tails is “homozygous alternate,” see Fig. 2b), each coin flip is one read, the sequencer makes mistakes ~1 % of the time, and saying which coin the referee selected is the same as calling the genotype. Figure 2b shows how the respective probabilities of each coin change with the number of flips, assuming the coins model a site with a minor allele frequency of 50 %. Before any flips have occurred, the probability of a heterozygous coin is 50 %, and the respective probabilities of the two homozygous coins are 25 %. However, the probabilities shift dramatically with only a few flips of the coin.

How many coin flips do you need to confidently call the identity of the coin? Clearly two flips are too few: for instance, after two heads (Fig. 2b-i), there is still a nearly 20 % chance of a heads–tails coin. Even the observation of a heads and a tails is insufficient to conclude a heads–tails coin, since the nonzero rate with which the referee lies means that there is still a 1 in 27 chance of the coin being heads–heads (Fig. 2b-ii) rather than heads–tails (Fig. 2b-iii).

Although two flips are obviously insufficient, with only 20 flips it is possible to achieve a remarkable 99.9999 % confidence in the coin’s identity; put differently, the chance of being wrong with 20 flips is less than one in a million. As expected, the chances of being wrong continue to drop with more coin flips. In fact, with 50 heads and no tails, the chance of the coin not being a heads–heads coin is 1 in 10^15^, which means that every person on Earth would call nearly 150,000 coin identities correctly before even one coin is miscalled.

The coin-flipping analogy casts important light on interpreting both data and marketing materials from NGS-based tests: since a SNP call is all but certain at 50× depth, extremely high read depth has an arguably bigger effect on increasing the cost of a test than improving its clinical performance.

Indeed, Fig. 2c shows that in an NGS test with 100× average depth, the vast majority of sites have >50× depth, which allows the test to generate conspicuously obvious and statistically significant genotype calls with extremely low error rates. The figure further shows that despite an average depth of 100×, the depth at many sites differs considerably from the average value, raising the question of which metric—average or minimum depth—is the best indicator of a test’s variant-call confidence. We revisit the coin analogy to gain insight: Suppose you have to correctly identify three different coins in succession and can select either an average of 50 flips per coin or a minimum of 20 flips per coin. Remarkably, the minimum of 20 flips is easily the best option here, since an unscrupulous referee could achieve an average of 50 by flipping the first coin 148 times and the other two coins only once each. Indeed, since read depth is not constant across all sites in NGS data (Fig. 2c), we suggest that minimum depth—not average depth—is the most direct and informative metric for assessing the confidence in a test’s variant calls.

One last important point about identifying short variants like SNPs and indels is that sequencer error is not uniform at all sites. Indeed, the NGS machine generally makes mistakes only 0.1 % of the time [22], but there are rare sites where the sequencer is systematically more error prone (e.g., ~1 % error rate). At these sites, the sequencer behaves like a referee who lies pathologically about coin-flip outcomes. Increasing the number of coin flips alone may not solve the problem, since each new result could still be a lie; similarly, increased sequencing depth alone may not yield good calls at sites corrupted by systematic error. However, you could outsmart the referee by having him repeatedly flip double-headed coins: If he reports back tails more often than the expected rate of random error, he is a liar. In an NGS context, this approach entails measuring thousands of reads at every site from reference samples that have well-established genotypes; sites with reproducibly elevated non-reference read counts, therefore, can be flagged for special handling (e.g., assessing the site with a different assay if the NGS errors prohibit sufficient call confidence).

## How are Large Deletions/Duplications Revealed from NGS Data?

Disease-causing mutations span a range of lengths: SNPs affect single bases and indels usually affect fewer than five bases, but del/dups can span hundreds to many thousands of bases. Unlike SNPs and indels—which are far shorter than NGS reads and thus are conspicuous within single reads—del/dups can far exceed an NGS read length. Nevertheless, del/dups can still be confidently identified from NGS data with the proper analysis strategy.

**Fig. 3:**
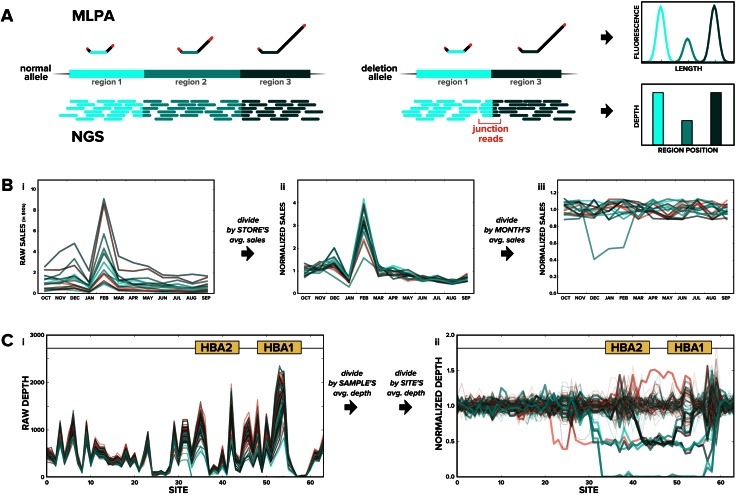
Del/dup calling from NGS data requires simple and intuitive processing of raw data. **a** Schematic of MLPA (*top*) and NGS (*bottom*) data for a sample in which one chromosome is normal and the other has a deletion. MLPA probes have a genome-binding sequence (*shades of green*), stuffer sequence to give them unique length (*black*), and binding sites for common primers (*red*) at the termini that enable multiplex amplification. For NGS, read depth can be pooled across a region (as depicted) or counted at a single site. In addition to depth data supporting a del/dup, NGS provides evidence of junction reads that further support the observation of a del/dup. For clarity, the ligation step that fuses two DNA fragments into the probes depicted in the figure is omitted. **b** A chocolate store that underperforms relative to others is revealed by dividing (*i*) the hypothetical annual sales volume for each store by its average (yielding *ii*) and then dividing once more by the monthly average across stores (giving *iii*). **c** Multiple samples with del/dups in the HBA locus are discovered by normalizing (*i*) the raw depth data across many sites by the sample average and then by the site average (yielding *ii*, where del/dup samples have *thick traces*)

To underscore the ability of NGS to call del/dups, we compare it to one of the current standards for clinical del/dup detection: multiplex ligation-dependent probe amplification (MLPA) [23]. Just as NGS has strong conceptual parallels with Sanger sequencing, NGS calling of del/dups has much in common with MLPA. In a single MLPA experiment, the copy number of ~40 locations in the genome can be assessed. Each location is bound by a probe whose length is specific to that location (Fig. 3a). Probes that successfully bind to genomic DNA are competent for amplification, thus the amount of amplified probe is proportional to the amount of genomic DNA (i.e., a deletion that halves the amount of genomic DNA will yield half as much amplified probe). Probes are fluorescently labeled, separated by size in an electric field, and measured with a fluorescence detector using the same instrument employed for Sanger sequencing. A duplication or deletion in MLPA manifests as one or more genomic locations—each with a different peak in the MLPA data profile—having 50 % more or less of the expected probe abundance, respectively.

The above description of MLPA can effectively be summarized as follows: it measures the abundance of genomic DNA at a handful of locations. By comparison, because NGS inherently measures the abundance of DNA at millions of locations in the genome—where abundance is simply the read depth—it is particularly well suited to calling del/dups anywhere in the genome. Beyond its ability to probe far more sites, NGS is also superior to MLPA in its ability to identify del/dup junctions—e.g., between regions 1 and 3 in the deletion allele in Fig. 3a—which provide extremely compelling evidence for a large rearrangement. Although MLPA can measure a handful of hypothesized junctions with custom probes, an NGS experiment needs no special treatment to yield junction reads and thus could identify del/dup interfaces anywhere in the genome.

Unlike SNP and indel calling—where the raw reads themselves reveal a mutation—del/dup detection using NGS requires numerical processing of the data before the variants become conspicuous. We again use an analogy to provide intuition for the approach. Suppose you are the new regional manager for a chocolate-retail company. You want to find which stores have had deflated sales numbers for a sustained period, so that you can best deploy resources to help them.

Figure 3b-i shows last year’s raw sales numbers by month for the 15 stores in your region. A few important features are clear from this plot: (1) stores with high sales—perhaps those in choice locations with lots of chocolate enthusiasts—tend to have high sales across each month, (2) all chocolate stores have spiky sales, with expected peaks in December for the holidays and in February for Valentine’s Day, and (3) it is nearly impossible to tell from this plot which stores, if any, are underperforming. Fortunately, you can resolve the last point by addressing the first two in turn.

To account for the fact that some stores have higher baseline sales than other stores, divide each store’s monthly sales by its yearly average, yielding Fig. 3b-ii. Now all stores are operating from the same effective baseline, but it is still not obvious which store is struggling due to the monthly sales spikes. Thus, to mitigate the monthly sales spikes that affect all stores, calculate the average adjusted sales across stores for each month (e.g., February’s adjusted average is 3), and divide all adjusted sales numbers by their corresponding monthly average (i.e., divide all stores’ February sales by 3). This second step yields the data in Fig. 3b-iii, where the struggling store is readily apparent.

This chocolate-store example has very strong similarities with del/dup analysis from NGS data. The chocolate analogy applied two simple normalizations to bulk sales data collected across many stores and many months to reveal a single store with depleted sales. By comparison, because depth in most NGS applications is proportional to the relative copy number of DNA in a region, the same two normalizations can be applied to depth data across many samples and many sites to discover a sample with a del/dup. Figure 3c-i shows raw NGS data for 96 samples across 60 sites in the HBA region on chromosome 16 that can cause alpha-thalassemia when gene copy number is disrupted [14] (note that since del/dup detection depends on relative changes in read depth, it is useful to have ≥50× minimum depth in such regions). There are eight carrier samples and three affected samples in the plot, yet none are apparent from the raw data. Some samples have a higher baseline because their DNA was more efficiently amplified, and some probes perform better than others at capturing DNA for sequencing. However, by applying the chocolate-store approach of normalizing by each sample’s baseline depth and then normalizing again by each site’s adjusted average, we generate a plot (Fig. 3c-ii) in which the samples with del/dups are easily and confidently identified.

## Conclusion

In this report, we have described how NGS data are collected and analyzed. We showed that the mechanism of NGS is not a fundamental departure from its predecessor, but rather an improved and scaled version of Sanger sequencing that allows for a staggering increase in data quality and throughput. We argue that minimum depth is a better reflection of a test’s variant-call confidence than average depth, and demonstrate that SNPs, indels, and del/dups can be confidently identified using intuitive analysis techniques. Our primary hope is that we can make NGS-based genetic tests more accessible to patients by making the inner workings of the technology itself more accessible to practitioners of genetic medicine.
